# Eosinophils at diagnosis are elevated in amyotrophic lateral sclerosis

**DOI:** 10.3389/fneur.2023.1289467

**Published:** 2023-12-21

**Authors:** Jing Yang, Tingting Liu, Lei Zhang, Xin Li, Feng Ping Du, Qi Liu, Hui Dong, Yaling Liu

**Affiliations:** ^1^Department of Neurology, The Second Hospital of Hebei Medical University, Shijiazhuang, China; ^2^The Key Laboratory of Neurology (Hebei Medical University), Ministry of Education, Shijiazhuang, China; ^3^Neurological Laboratory of Hebei Province, Shijiazhuang, China; ^4^Department of Emergency, The Second Hospital of Hebei Medical University, Shijiazhuang, China

**Keywords:** amyotrophic lateral sclerosis (ALS), ALS functional rating scale-revised (ALSFRS-R), peripheral blood leukocyte, eosinophils, lymphocyte to eosinophils ratio (LER)

## Abstract

**Introduction:**

Amyotrophic lateral sclerosis (ALS) is a rare, devastating neurodegenerative disease that affects upper and lower motor neurons. To date, no effective treatment or reliable biomarker for ALS has been developed. In recent years, many factors have been proposed as possible biomarkers of ALS; however, no consensus has been reached. Therefore, a reliable biomarker is urgently needed. Eosinophils may play a crucial role in healthy humans and diseases, and serve as a biomarker for many chronic diseases.

**Methods:**

Routine blood test results were collected from 66 healthy controls and 59 patients with ALS. The percentages and total numbers of each cell population were analyzed, and the correlation between these indicators and patient ALS functional rating scale–revised (ALSFRS-R) score or disease progression rate (ΔFS score) was analyzed.

**Results:**

Compared to healthy controls, the number of blood leukocytes, neutrophils, monocytes, and basophils was significantly decreased in patients with ALS (*p* = 0.002, *p* = 0.001, *p* = 0.049, and *p* < 0.0001, respectively). There was an increase in the number of eosinophils (*p* < 0.0001), but no difference in the number of lymphocytes between patients with ALS and healthy controls was found (*p* = 0.563). Compared to healthy controls, the percentage of neutrophils was decreased and the percentage of lymphocytes and eosinophils was increased in patients with ALS (*p* = 0.01, *p* = 0.012, and *p* = 0.001, respectively). There was no difference between patients with ALS and healthy controls in the percentage of monocytes and basophils (*p* = 0.622 and *p* = 0.09, respectively). However, only the percentage and number of eosinophils had a correlation with the ΔFS score. Further multivariate analysis revealed a significant correlation between the disease duration, eosinophil count and percentage, and the disease progression rate (*p* < 0.0001, *p* = 0.048, and *p* = 0.023, respectively). The neutrophil-to-eosinophil ratio (NER), lymphocyte-to-eosinophil ratio (LER), and monocyte-to-eosinophil ratio (MER) were significantly lower in patients with ALS than in healthy controls. However, only the LER was significantly correlated with the ΔFS score.

**Conclusion:**

These observations implicate neutrophils, lymphocytes, and eosinophils as important factors, and increasing eosinophil counts were negatively correlated with the ΔFS score in patients with ALS.

## 1 Introduction

Amyotrophic lateral sclerosis (ALS) is a severe adult-onset neurodegenerative disease that primarily affects motor neurons, resulting in muscle atrophy, spasticity, hyperreflexia, and paralysis. Although the clinical presentation of the disease varies widely among patients, the median survival is 3–5 years from symptom onset (1). The pathophysiology of ALS includes oxidative stress, glutamate excitotoxicity, protein homeostasis, defects in RNA processing, impaired axonal transport, and mitochondrial dysfunction (2). Although riluzole (3) and edaravone (4) have been approved by the Food and Drug Administration (FDA), there is currently no cure or effective treatment for ALS.

Accumulating evidence indicates peripheral immune abnormalities exist in patients with ALS (5–8). Peripheral blood leukocyte includes neutrophils, monocytes, lymphocytes, eosinophils, and basophils. The total number of blood leukocytes, neutrophils, and natural killer (NK) cells was found to be elevated in patients with ALS, and the number of neutrophils was significantly correlated with disease progression; however, NK cells and T lymphocyte populations act as prognostic markers for survival (9). The percentage of neutrophils is significantly increased in patients with ALS. The neutrophil-to-monocyte (NMR) ratio correlates with disease progression (10), and the neutrophil-to-lymphocyte (NLR) ratio may be used as a prognostic biomarker for survival in patients with ALS (11). Different cells may play different roles during each phase of ALS, and there is a critical need to investigate the role of peripheral blood leukocytes in patients diagnosed with ALS. In the present study, we collected laboratory data and peripheral blood leukocytes of patients with ALS and examined the relationship between several cell populations in ALS with the disease progression rate (ΔFS score).

## 2 Materials and methods

### 2.1 Subjects

We reviewed the clinical information and collected laboratory data of blood leukocytes of patients with ALS who were diagnosed in the Department of Neurology of the Second Hospital of Hebei Medical University from January 2021 to October 2022. Information on the corresponding healthy controls and the laboratory data of blood leukocytes were collected from the physical examination department. All patients met the revised diagnostic criteria for ALS (12). The participants were first diagnosed with ALS, had not used riluzole and edaravone, and had not recently used hormone-based or anti-inflammatory drugs. Participants with a ventilator, tracheotomy, or acute or chronic inflammatory diseases, including acute pneumonia and rheumatoid arthritis, were excluded. All the participants provided written informed consent. The ALS functional rating scale (ALSFRS-R) was applied to assess the severity of the disease (13), and the rate of disease progression was evaluated using the ALSFRS-R score: progression rate ratio (Delta FS, ΔFS) according to the following formula: (48-ALSFRS-R score at the time of diagnosis)/time onset to diagnosis) (14). This study was approved by the ethical committee of the Second Hospital of Hebei Medical University (2022-R196).

### 2.2 Blood collection

Human venous blood was collected from patients with ALS and healthy controls in the morning after an overnight fast, between 6 and 7 am, from the cubital region using disposable venous blood collection devices (Sterile K2EDTA, Hebei Xinle Medical Equipment Technology Co.). Blood samples were analyzed using automatic hematological analysis (UniCel DxH 800 Coulter Cellular Analysis System, Beckman Coulter) in the laboratory.

### 2.3 Statistical analyses

Statistical analyses and graphing were performed using GraphPad Prism 9 (GraphPad software) and SPSS26. All data were presented as the mean ± SD, and the Shapiro–Wilk test was used to examine the data distribution. For normally distributed data, an unpaired *t*-test was used for analyzing the between-group differences, whereas Pearson’s correlation coefficient test was utilized to analyze the correlation. For abnormally distributed data, the Mann–Whitney *U* test was used for the differences analysis, and Spearman’s rank correlation coefficient test was used for correlation analysis. The multivariable regression model was adjusted for age, sex, and body mass index (BMI). *p*-values ≤0.05 were considered statistically significant.

## 3 Results

### 3.1 Demographic information of the participants

**Figure 1:**
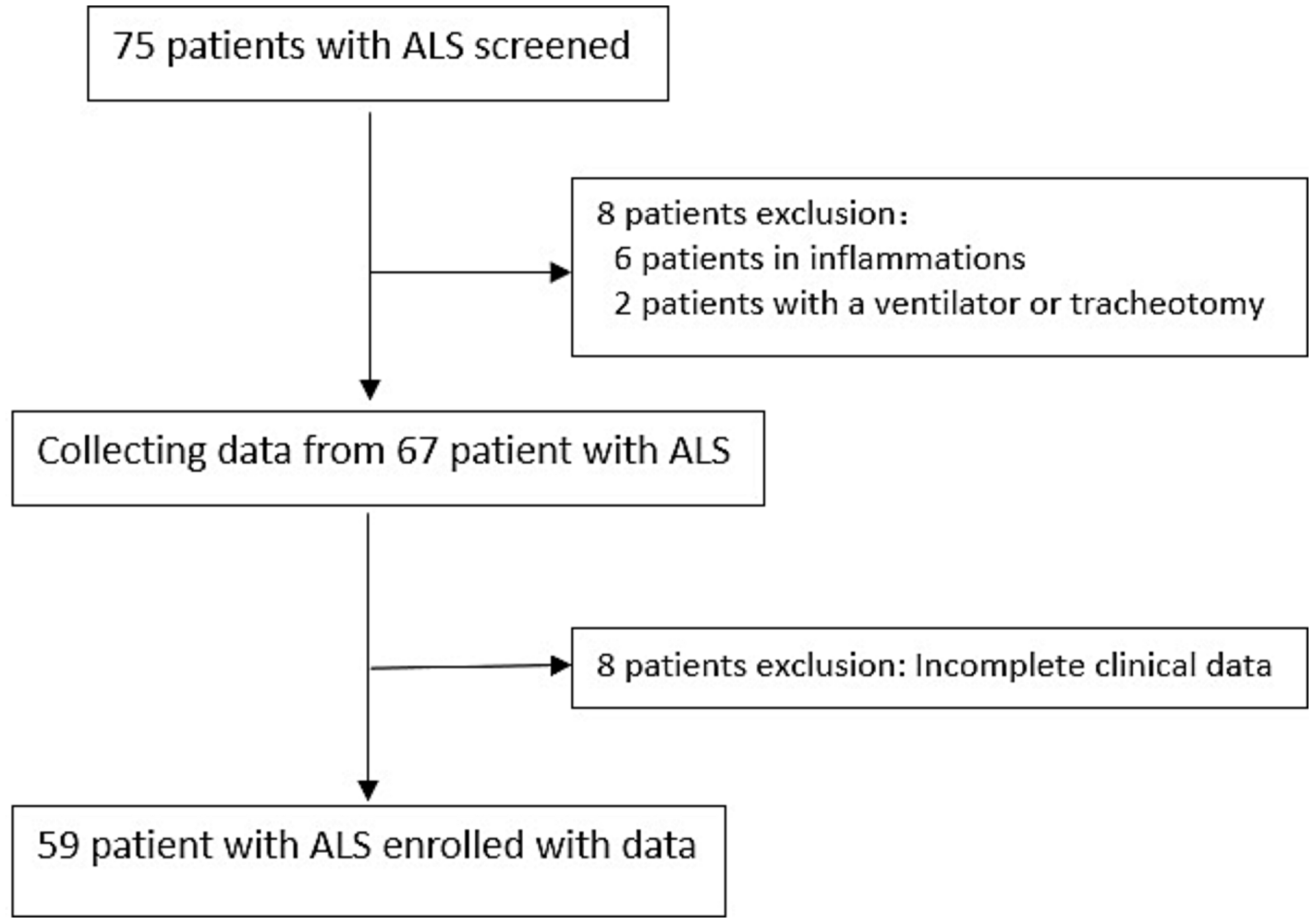
Screening protocol for patients with ALS.

Data from 59 of 75 patients diagnosed with ALS at the Second Hospital of Hebei Medical University between January 2021 and October 2022 were collected (Figure 1). The healthy control group consisted of 66 patients (40 men and 26 women) with a mean age of 55 ± 8 years and a mean BMI of 23.93 ± 2.164. The ALS group included a total of 59 patients (39 men and 20 women), with a mean age of 58 ± 10 years and a mean BMI of 23.42 ± 2.394. The distributions of age, sex, and BMI did not differ significantly between the two groups (Table 1).

**Table 1:** Demographic parameters of the control group and ALS patients.

	Healthy controls	Patients with ALS	*p*-value
No. of participants	66	59	
Age, mean (SD), y	55 (8)	58 (10)	>0.05
*Sex, No.*			
Females	26	20	
Males	40	39	
*Site of onset, No.*			
Bulbar	NA	23	
Limb	NA	36	
BMI, mean (SD)	23.93 (2.164)	23.42 (2.349)	>0.05
Disease duration, mean (SD), m	NA	12.36 (7.245)	
ALSFRS-R total score, mean (SD)	NA	37.39 (4.325)	
ΔFS	NA	1.212 (0.9243)	

BMI, body mass index; ALSFRS-R, ALS functional rating scale-revised; ALS, amyotrophic lateral sclerosis; ΔFS, (Delta FS ratio (rate of disease progression) = (48-ALSFRS-R score at time of diagnosis)/time onset to diagnosis).

### 3.2 Peripheral blood leukocyte levels are altered in patients with ALS

Following their isolation from peripheral blood, leukocytes, which include neutrophils, monocytes, lymphocytes, eosinophils, and basophils, from each patient were counted by an automated blood cell analyzer. The total number of blood leukocytes was significantly lower in patients with ALS than in healthy controls (*p* = 0.002). The number of lymphocytes in patients with ALS was not significantly different from that in healthy controls (*p* = 0.563), whereas neutrophils, monocytes, and basophils were decreased (*p* = 0.001, *p* = 0.049, and *p* < 0.0001, respectively), and eosinophils were increased in patients with ALS (*p* < 0.0001). Comparing the percentages of these cells with the leukocytes between the two groups, we discovered that the percentage of neutrophils in patients with ALS was decreased (*p* = 0.01), and the number of lymphocytes and eosinophils was increased (*p* = 0.012 and *p* = 0.001, respectively). However, the monocyte and basophil counts were not significantly different from those in healthy controls (*p* = 0.622 and *p* = 0.09, respectively) (Table 2).

**Table 2:** The number and percentage of peripheral blood leukocytes in patients with ALS and healthy controls.

	Healthy controls	Patients with ALS	*p*-value
Leukocytes (*10^9^/L)	6.628 ± 1.638	5.752 ± 1.66	0.002
Neutrophil (*10^9^/L)	4.187 ± 1.47	3.423 ± 1.28	0.001
Lymphocyte (*10^9^/L)	1.93 ± 0.727	1.864 ± 0.651	0.563
Monocyte (*10^9^/L)	0.418 ± 0.121	0.376 ± 0.135	0.049
Eosinophil (*10^9^/L)	0.052 ± 0.052	0.154 ± 0.106	<0.0001
Basophil (*10^9^/L)	0.118 ± 0.118	0.025 ± 0.025	<0.0001
Neutrophil (%)	61.46 ± 9.003	57.51 ± 9.247	0.01
Lymphocyte (%)	28.8 ± 8.37	32.44 ± 8.919	0.012
Monocyte (%)	6.732 ± 2.197	6.563 ± 1.943	0.622
Eosinophil (%)	1.898 ± 1.106	2.621 ± 1.512	0.001
Basophil (%)	0.467 ± 0.245	0.543 ± 0.294	0.09

### 3.3 Functional status and disease progression rate in patients with ALS

**Figure 2:**
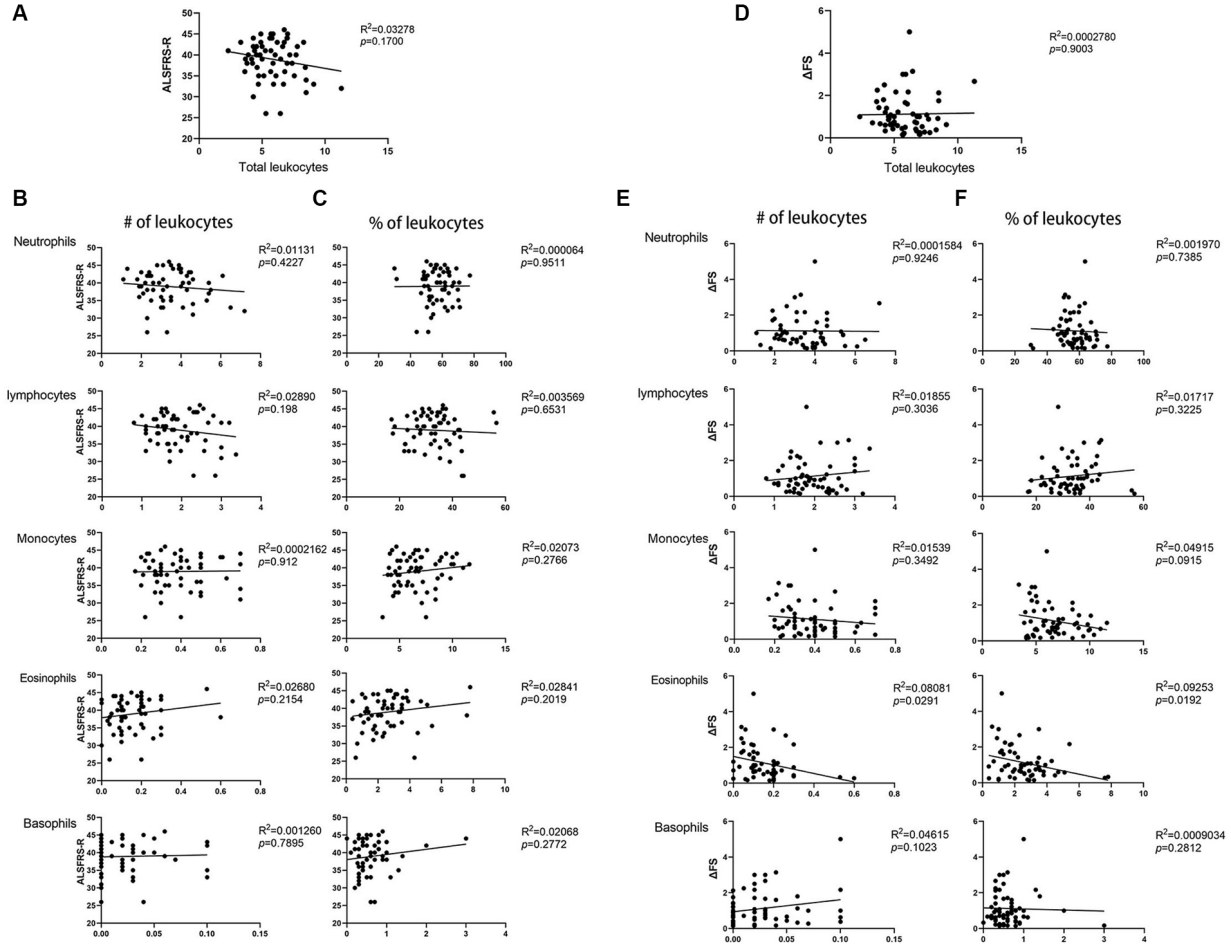
Correlation between the ALSFRS-R score or the disease progression rate (ΔFS) and peripheral blood leukocyte in patients with ALS. **(A)**The total number of leukocytes in patients with ALS showed no relationship with the ALSFRS-R score. **(B)** The number of neutrophils, lymphocytes, monocytes, basophils, and eosinophils in patients with ALS correlated with the ALSFRS-R score. **(C)** The percentage of neutrophils, lymphocytes, monocytes, basophils, and eosinophils in patients with ALS correlated with the ALSFRS-R score. **(D)** The total number of leukocytes in patients with ALS showed no relationship with ΔFS. **(E)** The number of neutrophils, lymphocytes, monocytes, basophils, and eosinophils in patients with ALS correlated with the ΔFS. **(F)** The percentage of neutrophils, lymphocytes, monocytes, basophils, and eosinophils in patients with ALS correlated with the ΔFS.

Based on the abovementioned results, we speculated that leukocyte and cell types in patients with ALS may be related to disease severity. The percentages and number of cell types were compared with patient ALSFRS-R scores, and we observed that the total number of leukocytes showed no relationship with the ALSFRS-R score (*p* = 0.17) (Figure 2A). The numbers and percentages of neutrophils and monocytes both decreased but did not correlate with the ALSFRS-R score (*p* = 0.4227, *p* = 0.9511, *p* = 0.912, and *p* = 0.2766, respectively). The numbers and percentages of eosinophils both increased, but they did not correlate with the ALSFRS-R score (*p* = 0.2154 and *p* = 0.2019, respectively). Similarly, we observed no correlation between lymphocytes or basophils counts and ALSFRS-R scores (*p* = 0.198, *p* = 6,531, *p* = 7,895, and *p* = 0.2772, respectively) (Figures 2B,C).

We then examined whether leukocytes and other cell types in patients with ALS are related to the disease progression rate. we observed no correlation between the total number of leukocytes and the disease progression rate (*p* = 0.9003) (Figure 2D). The number and percentage of neutrophils, lymphocytes, and basophils showed no relationship with the disease progression rate (*p* = 0.9246, *p* = 0.7385, *p* = 0.3036, *p* = 3,225, *p* = 0.1023, and *p* = 2,812, respectively). However, we observed that the number and percentage of eosinophils correlated with the disease progression rate (*p* = 0.0291 and *p* = 0.0192, respectively) (Figures 2E,F). Further multivariate analyses revealed a significant correlation between disease duration, eosinophil counts and percentages, and the disease progression rate (*p* < 0.0001, *p* = 0.048, and *p* = 0.023, respectively). These data indicate that eosinophils may play a role in ALS.

**Figure 3:**
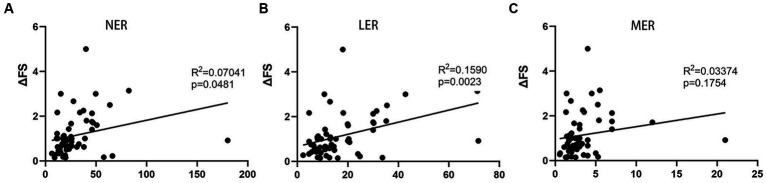
Correlation between progression rate (ΔFS) of ALS and LER, NER, and MER. **(A)** Correlation between ΔFS and NER. **(B)** Correlation between ΔFS and LER. **(C)** Correlation between ΔFS and MER. LER, lymphocyte-to-eosinophil ratio; NER, neutrophil-to-eosinophil ratio; MER, monocyte-to-eosinophil ratio.

To establish a more accurate correlation between leukocytes and ALS progression, we observed that NER, LER, and MER were significantly decreased in patients with ALS compared to healthy controls (*p* < 0.0001, *p* = 0.0009, and *p* = 0.0002, respectively) (Supplementary Figure S1). Therefore, we simultaneously calculated the NER, LER, and MER for each patient at the same time. A relationship between the NER or LER and ΔFS score was found, with the LER exhibiting a more significant association than the NER (Figures 3A,B). However, the MER and ΔFS score were not correlated (Figure 3C). Further multivariate analysis revealed significant correlations among disease duration, the LER, and disease progression rate (*p* < 0.0001 and *p* = 0.023, respectively). These data suggest that the LER may be used to predict the rate of disease progression in patients with ALS.

## 4 Discussion

The present study investigated the role of peripheral blood leukocytes in patients with ALS; specifically, we wanted to examine whether leukocytes and other peripheral cell types in patients with ALS are related to the disease progression rate. Additionally, our goal was to identify a potential biomarker that can be obtained from a simple routine blood test, offering a means to monitor changes in the disease. Although many factors (11, 15–18) have been proposed as potential biomarkers in ALS, there is still no consensus. Peripheral blood leukocytes are used to analyze the role of peripheral immune cells in patients with ALS, mainly because peripheral blood leukocytes are easily obtained and monitored, and the operation does not affect the central nervous system (CNS). In addition, several previous studies (5–8, 11) have reported that peripheral immune cells are involved in the pathogenesis of ALS.

Consistent with previous reports (9–11), we observed that peripheral blood leukocyte levels were altered in patients with ALS compared with healthy controls. The number of blood leukocytes, neutrophils, monocytes, and basophils is decreased, and the number of eosinophils is increased in patients with ALS. However, we observed that the percentage of neutrophils decreased and the percentage of lymphocytes and eosinophils increased in patients with ALS compared with healthy controls. These results are not consistent with those of previous studies, in which the number of peripheral blood neutrophils increased in patients with ALS (10, 11, 19, 20). We hypothesize that the differences in these results may be related to the experimental methods. In this study, the number of peripheral blood leukocytes was counted using an automated blood cell analyzer, however, in previous studies, it was observed by flow cytometry. In a cohort study (21), leukocyte, neutrophil, and monocyte levels gradually increased over time, but there was no obvious temporal trend in lymphocyte levels. We observed that lymphocyte levels increased at the time of initial diagnosis, but unfortunately, we did not have follow-up data. These results further support the presence of immune abnormality in ALS.

To the best of our knowledge, our study is the first to pay attention to eosinophils, and we found that the number and percentage of eosinophils increased in patients with ALS compared with healthy controls, which was negatively correlated with the ΔFS score in patients with ALS. However, despite the significant *p*-values indicating a correlation between eosinophils and the disease progression rate, the low R2 values indicated a weak correlation between the two. Our results provide further support for the involvement of peripheral immune cells in the pathogenesis of ALS and suggest that eosinophils may be involved in ALS; however, the exact mechanism remains unclear. Eosinophils are peripheral blood leukocytes that are known to play a role in allergic diseases and infections; however, it is now recognized that eosinophils are multifunctional leukocytes containing amounts of granules that also play critical roles in immune responses, including metabolism regulation, neuronal regulation, microbiome regulation, and tissue development. Thus, based on the above research, eosinophils may play a crucial role in healthy humans (22, 23). In disease, cytokines, immunoglobulins, and complements can trigger eosinophil production, leading to the secretion of proinflammatory cytokines (24). A recent study (25) showed that eosinophils in adipose tissue play an important role in metabolic diseases. In this study, we found that the number and percentage of eosinophils increased in patients with ALS compared to healthy controls, which may be related to the inflammatory reaction in patients with ALS. Previous research works (26–29) that have explored the role of peripheral immune cells in several diseases have focused on neutrophils, monocytes, and lymphocytes, whereas eosinophils have largely been ignored. An increasing body of research (23, 24, 30) has revealed that eosinophils may have a larger role in human health and diseases. Eosinophils are key regulators of perivascular adipose tissue and vascular functionality (31) and are involved in the browning of white fat (32). In this study, we observed that the number of eosinophils increased in patients with ALS compared with healthy controls, and combined with the abovementioned research, we speculated that eosinophils may play an important role in ALS and in adipose tissue. However, we did not observe alterations in eosinophil counts in adipose tissue in this study, and we do not yet know whether eosinophils play a role in adipose tissue in ALS. In addition, eosinophils in white adipose tissue, known as major interleukin-4 (IL-4) expressing cells, have been shown to play an important role in controlling the weight and glucose levels of mice fed a high-fat diet (33). Combined with previous research showing that a high-fat diet can prolong the survival time in SOD1G93Amice, this may be related to the activities of eosinophils.

Moreover, eosinophils are resident cells found in high numbers in the intestine and play an important role in maintaining the integrity of intestinal villi, intestinal function, and lipid absorption. Moreover, eosinophils play a critical role in facilitating mutualistic interactions between the host and the microbiota (34). Previous studies have shown that patients with ALS suffer from a flora imbalance and that the gut microbiome is of crucial importance in patients with ALS (35, 36). Overall, these results indicate that eosinophils may play an important role in patients with ALS.

Further evaluation of the NER, LER, and MER as potential indicators may prove useful in determining the disease progression rate of ALS at diagnosis. We found that the LER decreased in patients with ALS compared to healthy controls and that this ratio correlated with the rate of disease progression in patients with ALS. Various factors have been proposed as possible indicators of ALS, including the neutrophils-to-monocytes ratio (NMR) (10), serum C-reactive protein (CRP) (18), the neutrophil-to-lymphocyte ratio (NLR) (37), and IL-6 (38). However, to the best of our knowledge, this is the first study to report the LER as a possible indicator of ALS. These data support previous studies suggesting that immune cells in the blood are involved in the pathogenesis of ALS (9, 39). Further evaluation of the LER as a potential index may require further research and verification.

Eosinophil counts in ALS are elevated at diagnosis, although this finding is not without limitations. First, the main limitation of this study is the lack of follow-up data to assess the long-term effects of eosinophils on the prognosis of patients with ALS and changes in eosinophils over time. Second, this study did not provide a detailed analysis of the relationship between eosinophils and disease progression, nor did it rule out the influence of possible confounding factors, or explore the potential mechanisms. Third, the sample size of the study was relatively small, meaning that our data may not be fully representative, which may affect the generalizability of the findings. Fourth, the results were inconsistent with those of previous studies, particularly regarding the changes in peripheral blood leukocyte levels in patients with ALS. This may be due to differences in the experimental methods employed. Therefore, a larger sample size is needed to reliably comment on the role of eosinophils in the prognosis of patients with ALS. In subsequent experiments, we will follow patients over time to assess the long-term effect of eosinophils on the prognosis of patients with ALS as well as changes in eosinophils over time. To explore possible mechanisms and identify potential index targets, we will perform cellular and animal experiments in future studies.

In conclusion, our data demonstrate, for the first time, an important role for eosinophils in patients with ALS, further emphasizing the contribution of neutrophils, lymphocytes, and eosinophils to physiological processes. We found that the observed increase in eosinophils was negatively correlated with the ΔFS score in patients with ALS, suggesting that eosinophils were protective in patients with ALS. These findings provide additional evidence for the participation of peripheral immune cells in ALS and provide insights into peripheral immunotherapy in ALS. We will further verify these findings in our follow-up study.

## Data availability statement

The raw data supporting the conclusions of this article will be made available by the authors, without undue reservation.

## Ethics statement

The studies involving humans were approved by the Ethical Committee of the Second Hospital of Hebei Medical University. The studies were conducted in accordance with the local legislation and institutional requirements. The participants provided their written informed consent to participate in this study.

## Author contributions

JY: Data curation, Methodology, Writing – original draft. TL: Data curation, Methodology, Writing – original draft. LZ: Data curation, Investigation, Software, Writing – original draft. XL: Methodology, Software, Writing – original draft. FD: Data curation, Investigation, Writing – original draft. QL: Formal analysis, Project administration, Supervision, Writing – original draft. HD: Conceptualization, Project administration, Supervision, Writing – review & editing. YL: Conceptualization, Funding acquisition, Writing – review & editing.
