# Supplementary figures and images for: Eosinophils at diagnosis are elevated in amyotrophic lateral sclerosis
Source: Front Neurol. 2023 Dec 21;14:1289467. doi: 10.3389/fneur.2023.1289467 (PMC10768070; doi:10.3389/fneur.2023.1289467)

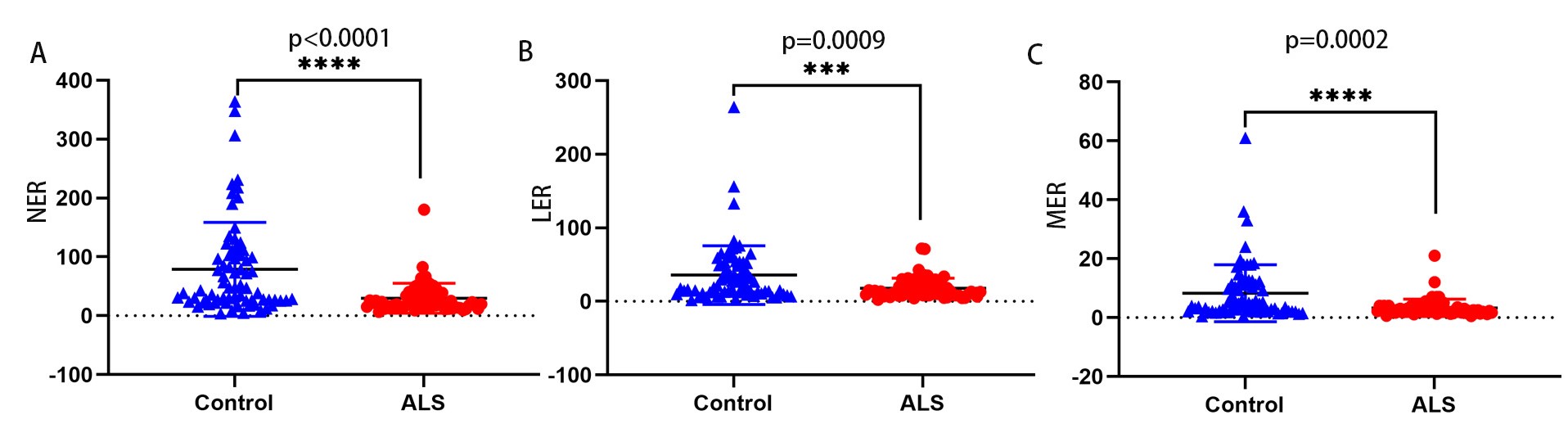

Supplement: Supplementary file 1 [file Image_1.JPEG]
